# Formability Assessment of C1100 Pure-Copper Tube Considering an Enhanced Modified Maximum Force Criterion

**DOI:** 10.3390/ma18091919

**Published:** 2025-04-24

**Authors:** Ngoc Anh Pham, Quoc Tuan Pham, Van Duy Dinh, Dac Trung Nguyen, Duc-Toan Nguyen, Tran Duc Hoan, Lai Dang Giang

**Affiliations:** 1 School of Mechanical Engineering, Vietnam Maritime University, Hai Phong 180000, Vietnam; anhpn.vck@vimaru.edu.vn; 2 School of Mechanical Engineering, Hanoi University of Science and Technology (HUST), Hanoi 100000, Vietnam; tuan.phamquoc@hust.edu.vn (Q.T.P.); trung.nguyendac@hust.edu.vn (D.T.N.); 3 Faculty of Mechanical Engineering, Le Quy Don Technical University, Hanoi 100000, Vietnam; tranduchoan@lqdtu.edu.vn (T.D.H.); danggiang248@lqdtu.edu.vn (L.D.G.)

**Keywords:** C1100 copper tubes, forming limit diagram (FLD), MMFC2 model, tube expansion test, finite element analysis (FEA)

## Abstract

The Modified Maximum Force Criterion (MMFC) and Marciniak–Kuczynski (MK) models were initially developed to evaluate strain localization in sheet metals. This study investigates their use in predicting the forming limit diagram (FLD) of a tubular material, specifically C1100 pure-copper tubes. To achieve this, uniaxial tensile tests were performed to develop a robust constitutive model, capturing the material’s hardening behavior with a combined Swift–Voce hardening law. A MATLAB code was then developed to theoretically predict the FLD using an enhanced MMFC model, termed MMFC2, alongside the established MK model. These predictions were validated against experimental results from tube expansion tests. Additionally, the theoretical FLDs were integrated into finite element simulations of the tube expansion test to forecast tube bursting behavior. The comparisons reveal that the MMFC2 model’s predictions align more closely with experimental outcomes than those of the MK model, highlighting MMFC2’s superior potential for predicting FLDs in tubular materials.

## 1. Introduction

Tube hydroforming (THF) is an advanced metal-forming process that utilizes hydraulic pressure to deform tubular blanks into complex shapes. This technology finds extensive applications across various industries, including automotive, aerospace, defense, and consumer goods, owing to its uses in producing lightweight and high-strength components [1,2,3,4]. However, predicting tube bursting and failure during THF processes is critical in ensuring production efficiency and optimizing process parameters [5,6,7]. To address this, various theoretical and numerical approaches have been developed to predict failure mechanisms during THF.

Several researchers have proposed models for simulating strain localization and necking behaviors in tubular materials. For instance, Kim et al. [8] employed Swift’s instability criterion combined with Oyane’s damage model to predict necking in tubular materials. Similarly, Butcher et al. [9] integrated the Gurson–Tvergaard–Needleman (GTN) damage model into LS-DYNA simulations to effectively forecast tube bursting in DP600 steel tubes. Yuenyong et al. [10] further demonstrated the applicability of damage-based models by predicting failure in stainless-steel 304 tubes during THF operations.

Among the existing methods, the forming limit diagram (FLD) is often preferred for evaluating strain localization and failure during THF processes due to its simplicity [11,12]. In this regard, the Marciniak–Kuczynski (MK) method [13,14] is the most widely used approach for predicting FLDs in sheet metals. For tubular materials, this model has been extensively adopted to forecast failures in THF processes. Dong et al. [15] employed the MK method to estimate the fracture strains of AA6061 tubes. In their study, the value of the geometrical imperfection was determined from a numerical simulation of a uniaxial tensile test. Hashemi et al. [16] further enhanced the traditional MK model by incorporating groove rotation during deformation. They reported a strong agreement between the MK predictions and experimental data for AA6063 and AA6065 extruded tubes. Additionally, various ductile damage models have been integrated into the MK framework to identify the initiation of strain localization [17]. Despite its widespread use, the MK model’s predictions are significantly influenced by the chosen imperfection value and the constitutive equations used for the tubular materials. The determination of the imperfection parameter remains ambiguous, often relying on the modeler’s expertise. Moreover, the adoption of advanced constitutive models for tubular materials pose ongoing challenges in this field [18,19].

In addition to the MK method, the Modified Maximum Force Criterion (MMFC), introduced by Hora and colleagues [20], has gained significant attention in recent years. Various researchers have explored the MMFC to predict strain localization in a range of automotive sheet metals [21,22,23,24]. These studies highlight the potential of the MMFC in theoretically determining the FLD for diverse engineering materials. Building on the original approach, Pham et al. [25] recently proposed an enhanced condition for strain localization and introduced an improved variant called MMFC2. This updated model has demonstrated further accuracy in predicting the FLDs of several sheet metals. However, despite its advancements, the application of MMFC or MMFC2 to tubular materials remains unexplored, limiting their broader practical utility in manufacturing processes.

This study introduces a novel application of the MMFC2 model to predict FLDs for C1100 pure-copper tubes, a material widely used in industrial applications but under-explored in failure prediction research [26,27,28]. A hybrid numerical–experimental approach is adopted, involving uniaxial tensile tests to calibrate a material model based on the linear Swift–Voce (LSV) hardening law. The calibrated model is incorporated into the MMFC2 framework to predict the FLD, with tube expansion tests conducted to experimentally confirm the theoretical findings. Comparisons between the predictions of MMFC2 and MK models are provided to demonstrate the superior precision of the former to distinguish safe and fracture regions. From a theoretical perspective, discussions are provided to clarify the reasons behind the differences in these predicted FLDs. Moreover, finite element (FE) simulations of the failures observed in the tube expansion test offer concrete evidence to support the theoretical discussions. This research not only bridges a critical gap in the application of MMFC2 for tubular materials but also provides a robust framework to improve the prediction of failure during THF processes.

## 2. Experimental Procedure

This section details the experimental procedure carried out in this study. Uniaxial tensile tests were performed to calibrate a proper material model for the material tested. In addition, tube expansion tests are carried out to validate the theoretical predictions of the FLD.

### 2.1. Tested Material

The experiments were conducted using C1100 pure-copper tubes (as-received commercially in Kojako Vietnam, Hochiminh, Vietnam), a material commonly employed in electronics, civil construction, and mechanical engineering applications. This material was chosen due to the lack of comprehensive studies on its formability in the existing literature. In the past, the formability of copper materials has been investigated in various forms, such as multi-layer sheets [29,30,31,32], thick copper sheets [33], and bi-layer tubes [34]. The tubes had an outer diameter of 22 mm and a wall thickness of 1.0 mm. To enhance their ductility for testing, the tubes underwent a heat treatment process in accordance with the ASTM B280 standard [35]. Specifically, the tubes were heated to 600 °C, held at this temperature for 30 min, and then air-cooled to achieve the desired softening effect.

### 2.2. Uniaxial Tensile Test

**Figure 1:**
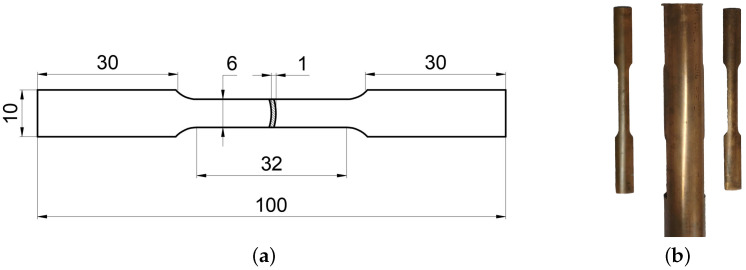
Samples used in the uniaxial tensile tests: (**a**) sample geometry and (**b**) uniaxial tensile samples.

The uniaxial tensile tests were conducted following the ASTM E8 [36] to characterize the hardening behavior of C1100 copper tubes. Samples were cut from a tube using a wire-cutting machine to ensure uniformity and consistency. Figure 1a illustrates the standard tensile sample geometry, while Figure 1b depicts the actual specimens. Tests were carried out on an MST-E45 universal tensile machine at a constant speed of 2 mm/min. During a test, the loading forces were measured by a load-cell and the displacements of the upper clamper were recorded to derive the stress–strain curves.

**Figure 2:**
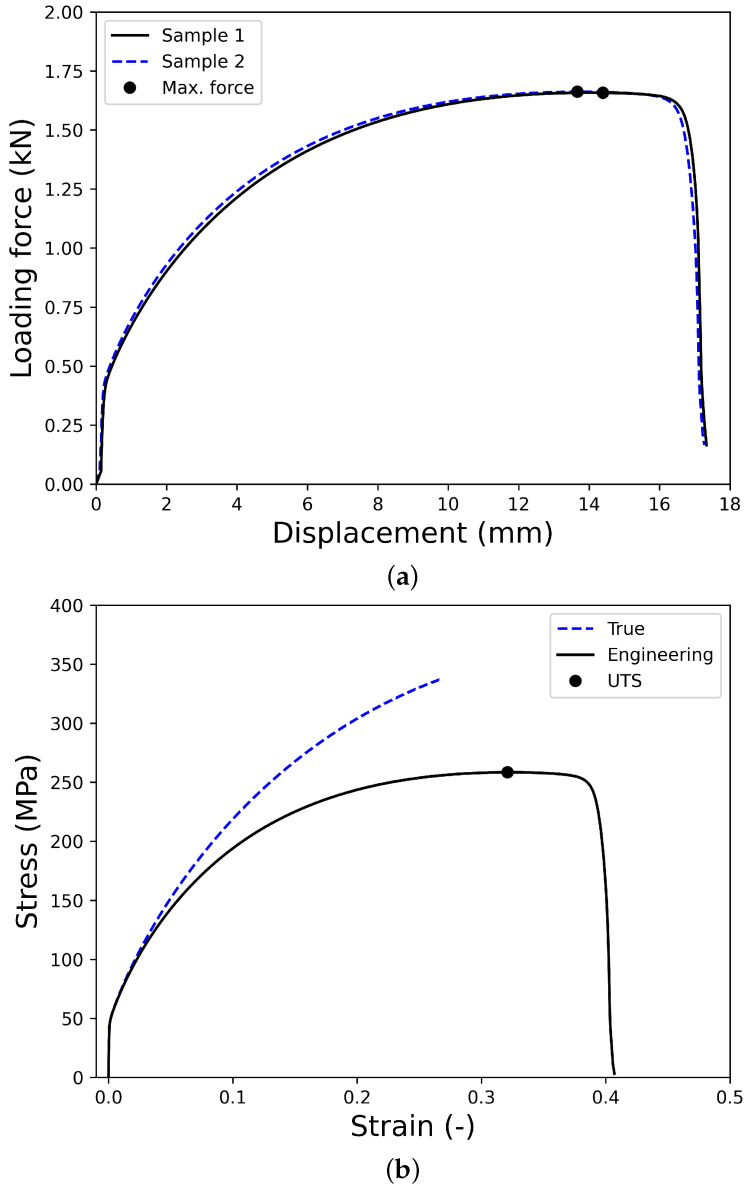
Results obtained from uniaxial tensile tests: (**a**) force–displacement curves and (**b**) stress–strain curves of the sample 1.

The force–displacement curves (Figure 2a) from two samples exhibited remarkable consistency, demonstrating the repeatability of the test setup. The stress–strain curves (Figure 2b) revealed significant strain hardening behavior, with the yield stress increasing from 50 MPa to 340 MPa, followed by continuous deformation until fracture beyond the ultimate tensile strength (UTS). The pronounced hardening characteristics highlight the material’s ductility and mechanical resilience, which are crucial for accurate FLD predictions. This uniaxial tensile test procedure provided essential input parameters for the LSV hardening law, which was incorporated into the FEA model and MATLAB-based (version R2017b) MMFC2 prediction framework. The consistency in the experimental results validated the accuracy of the material model calibration, showcasing the novelty of integrating precise mechanical characterization with theoretical FLD prediction for tubular materials.

### 2.3. Tube Expansion Test

**Figure 3:**
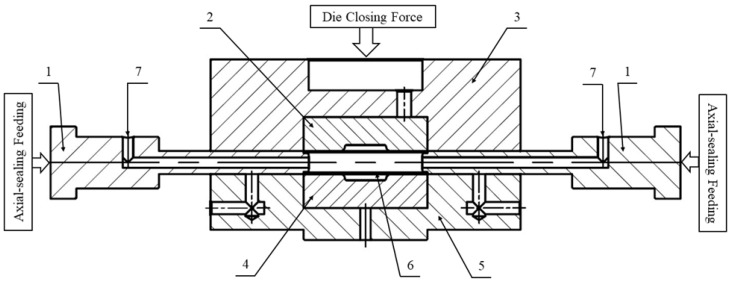
Drawing of the die set used in the tube expansion. Part list: 1—axial-sealing punch; 2—upper die; 3—upper die holder; 4—lower die; 5—lower die holder; 6—tubular blank; 7—fluid inlet.

A series of tube expansion tests were conducted to evaluate the formability of the tested material under varying stress states. In these experiments, tube blanks measuring 120.0 mm in length were subjected to a combination of increasing internal hydraulic pressure and axial compressive force. Figure 3 illustrates a sketch of the die set used in the experiments. The tube blank was placed within the working area, and two axial-sealing punches were gradually advanced inward to seal its ends, as shown in this figure.

The upper section of the die, consisting of the upper die and upper holder, was mounted on the upper platen of a YH-28 100 ton hydraulic press. A hydraulic force of up to 20 tons was applied to close the die. Hydraulic fluid was pumped through inlets in the axial-sealing punches to fill the interior of the tube blank. The axial-sealing punches were then advanced further to compress the tube blank from both ends. Meanwhile, the internal pressure was increased to the target levels of 18 MPa, 25 MPa, and 30 MPa. The tube blanks deformed and bulged until failure occurred.

**Figure 4:**
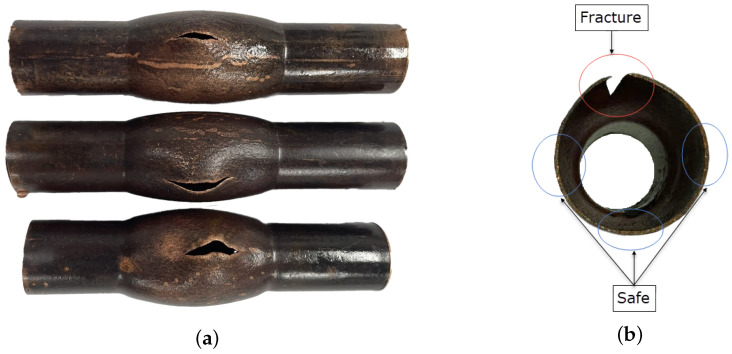
Deformed samples: (**a**) three samples subjected to the maximum internal pressure of 180 bar and (**b**) safe and fracture regions.

After the tests, the strain states of the deformed specimens were analyzed in two regions: (1) near the rupture, representing the “fracture” region, and (2) on the opposite side of the rupture, representing the “safe” region. Figure 4a shows an image of deformed specimens from tests conducted at an internal pressure of 180 bar, while Figure 4b highlights examples of the safe and fracture regions.

**Figure 5:**
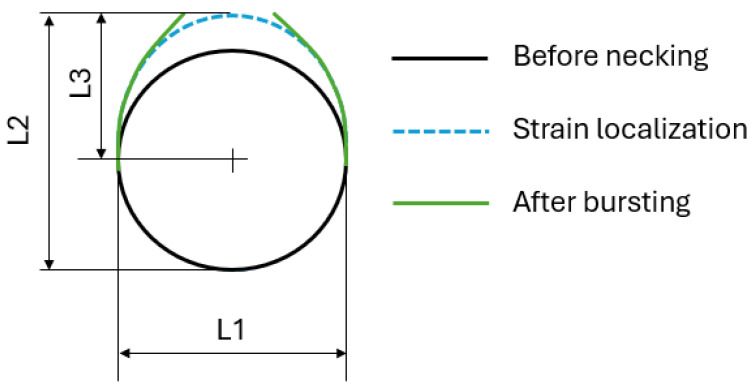
Determination of the strain state of the “safe” and “fracture” regions.

According to Figure 5, the strain states of the safe region are determined straightforwardly. Let us assume that the cross-section of the safe region is a circle. The diameter of the deformed tube prior to the strain localization is roughly estimated by the measured distance, $L_{1}$ in Figure 5. Hence, the circumferential strain of the safe region, $\varepsilon_{cir}^{safe}$, can be determined as follows:

(1) $$\varepsilon_{cir}^{safe}=\ln\left(\frac{L_{1}}{D_{0}}\right)$$

where $D_{0}$ denotes the initial outer diameter of the sample. In addition, the thickness strain, $\varepsilon_{t}^{safe/fract}$, is directly determined from the measured and initial thicknesses of the investigated regions. Subsequently, the longitudinal strain, $\varepsilon_{l}^{safe/fract}$, can be determined using the assumption of volume constant, for example:

(2a) $$\varepsilon_{cir}^{safe}+\varepsilon_{t}^{safe}+\varepsilon_{l}^{safe}=0$$

(2b) $$\varepsilon_{cir}^{fract}+\varepsilon_{t}^{fract}+\varepsilon_{l}^{fract}=0$$

Determination of the strain state at the onset of bursting is more challenging. Let us assume only half of the tube is deformed at the onset of strain localization and bursting as well, while the other half of the tube is unloading and remains the circular shape. Figure 5 illustrates the assumption with the largest distance from the bursting position, $L_{2}$. Therefore, the circumferential strain of the element located at the bursting region, $\varepsilon_{cir}^{fract}$, is calculated as:

(3) $$\varepsilon_{cir}^{fract}=\ln\left(\frac{L_{3}}{D_{0}/2}\right)=\ln\left(\frac{L_{2}+\left(L_{2}-L_{1}\right)}{D_{0}}\right)$$

Later, the strain state of the fracture region is determined by considering the assumption of volume constant.

For sheet metals, experimental FLDs can be established through tests adhering to standards like ISO-12004 [37]. However, no such standard exists for determining the FLD of tubular materials. According to the ISO-12004 standard, FLD determination requires measuring strain distribution along a section perpendicular to the fracture band. The task is feasible with modern techniques such as Digital Image Correlation [38] or Grid Analysis [39]. In this study, where these methods are unavailable, Equations (1) and (3) offer an approximate boundary for necking strains, defined by the safe and fracture strain limits. These experimental results will be compared with the theoretical FLDs predicted by the MMFC2 and MK models in the following section.

## 3. Theoretical Prediction of the Forming Limit Diagram

### 3.1. Constitutive Modeling

#### 3.1.1. Constitutive Equations

A constitutive model contains a system of equations governing the plastic deformation of the material under external loads. For metallic materials, the condition for plastic deformation is expressed as follows:

(4) $$F=\bar{\sigma }\left(\sigma \right)-H\left(\bar{\varepsilon }\right)\leq 0$$

where $\bar{\sigma }\left(\sigma \right)$ denotes a yield function, and $H(\bar{\varepsilon })$ denotes a hardening law. If F<0, then the tested material is subjected to elastic deformation. Otherwise, the plastic deformation occurs. Under plastic deformation, an associated flow rule is assumed to determine the directional plastic strain vector, of which the formula is expressed as:

(5) $$d\varepsilon_{ij}^{p}=d\bar{\varepsilon }\frac{\partial \bar{\sigma }}{\partial \sigma_{ij}}$$

**Figure 6:**
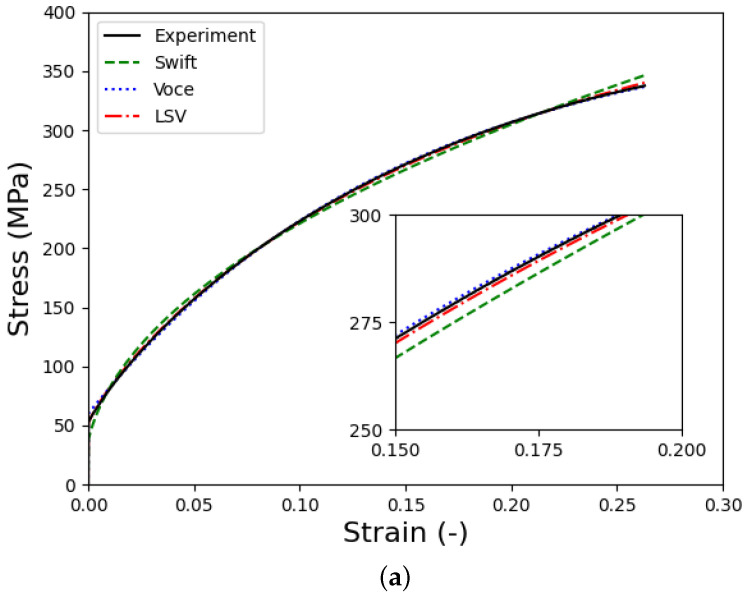

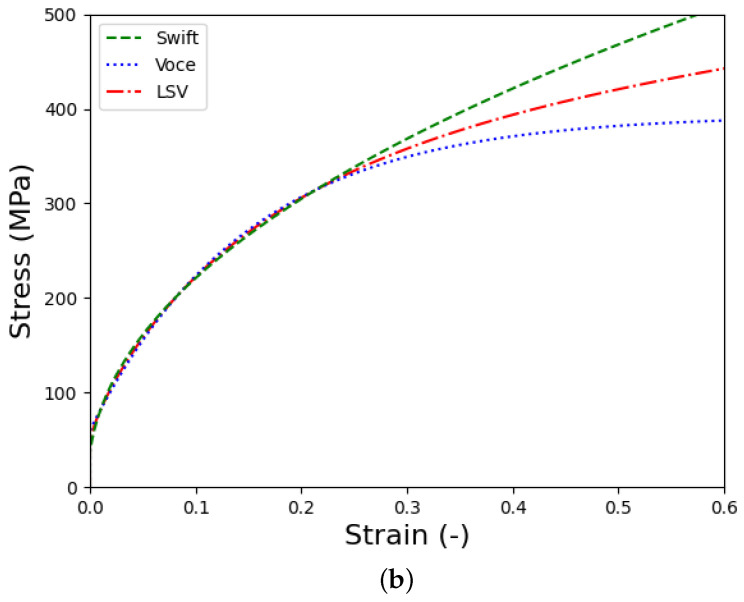
Identified hardening laws for the tested material: (**a**) fitting results and (**b**) extrapolation.

In sheet metal forming, several hardening laws, including the Swift, Voce, and their linear combination (LSV) models, are commonly used to describe the stress–strain relationship. These laws are integral in capturing material behavior during plastic deformation. Their formulas are expressed as follows:

(6) $$\mathrm{Swift}:\;H\left(\bar{\varepsilon }\right)=C{\left(\bar{\varepsilon }+\varepsilon_{0}\right)}^{n}$$

(7) $$\mathrm{Voce}:\;H\left(\bar{\varepsilon }\right)=A-B\exp\left(-k\bar{\varepsilon }\right)$$

(8) $$\mathrm{LSV}:\;H\left(\bar{\varepsilon }\right)=\alpha \left[C{\left(\bar{\varepsilon }+\varepsilon_{0}\right)}^{n}\right]+\left(1-\alpha \right)\left[A-B\;exp\left(-k\bar{\varepsilon }\right)\right]$$

where $C,\;\varepsilon_{0},\;n$ in the Swift model, A, B, k in the Voce model, and α in the LSV model represent parameters that must be determined to characterize the behavior of the tested material. In this study, parameters of the Swift and Voce models were identified using the curve-fitting method available in Excel to minimize discrepancies between the models and experimental data. Moreover, the parameter α in the LSV model was identified iteratively by comparing the simulated loading force with the measured data. Details on the calibration of the parameter α will be discussed in the next subsection. Figure 6 illustrates a comparison of the identified hardening laws against the experimental results, highlighting their alignment. Table 1 details the calibrated hardening law’s parameters.

Following established studies [4,11,40], the isotropic von Mises yield function was adopted to characterize plastic deformation in C1100 copper. This criterion is widely recognized for its effectiveness in modeling the yielding behavior of ductile metals. This research advances constitutive modeling by integrating multiple hardening laws with experimentally validated parameters to enhance the prediction of forming limit diagrams (FLDs) for C1100 copper tubes. The adoption of the LSV model, supported by rigorous calibration and FE simulations, underscores the novelty and accuracy of the proposed modeling approach for tubular materials.

#### 3.1.2. Validation

**Figure 7:**
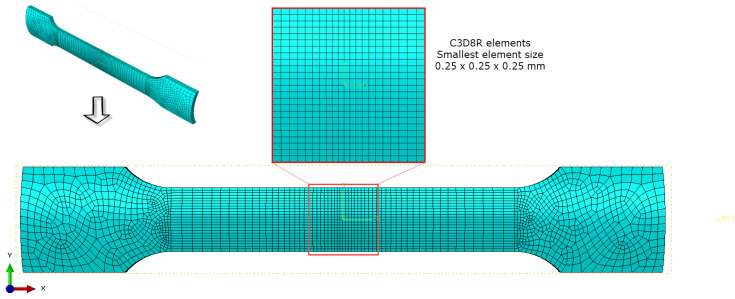
Sample modeling with C3D8R elements in Abaqus/Explicit software.

To validate the accuracy of the calibrated material models, a finite element (FE) model was created using Abaqus/Explicit software (version 6.13) to analyze the uniaxial tensile test. The blank was modeled with C3D8R solid elements, as depicted in Figure 7. The central region was meshed with elements sized at 0.25 × 0.25 mm. The element sizes were gradually increasing toward the ends of the sample. In addition, five layers were adopted to model the blank through its thickness. Three calibrated hardening laws were incorporated with the von Mises yield function to characterize the plastic deformation of the sample during the simulations. Consistent with the experimental setup, one end of the blank was fixed while the other was subjected to an axial displacement control of 18 mm during the simulation.

**Figure 8:**
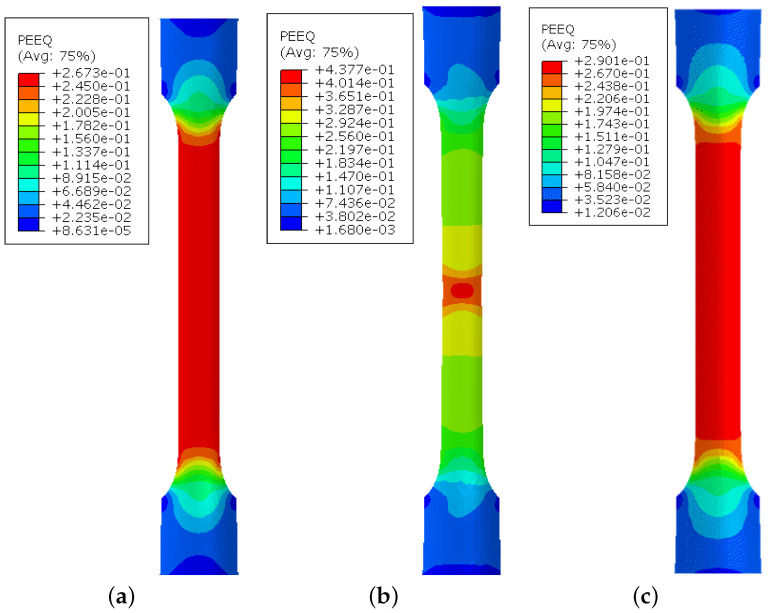
Prediction of the strain distribution on the outer surface of the deformed sample at the displacement of 13.2 mm: (**a**) Swift, (**b**) Voce, and (**c**) LSV.

**Figure 9:**
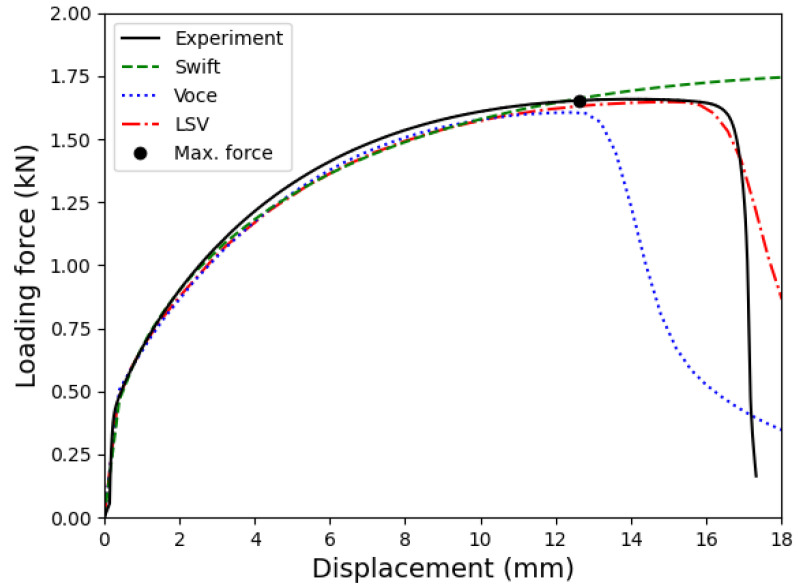
Comparison between the predicted loading forces based on different hardening laws and the measured data.

Figure 8 compares the strain distribution on the outer surface of the uniaxial sample predicted by three hardening laws at a displacement of 13.2 mm. The stage of comparison is the closest one to the instance of the maximum force, shown in Figure 9. As shown in this figure, the Voce hardening law predicted strain localization right after the maximum force with a maximum equivalent plastic strain PEEQ of 0.437. Meanwhile, the predictions of the Swift and LSV models are similar, where the deformation is distributed homogeneously. The maximum equivalent plastic strain PEEQ values predicted in these simulations at this stage are 0.267 and 0.29, respectively. In all simulations, values of PEEQ further exceeded the maximum strain measured during the uniaxial tensile test, demonstrating the importance of accurate prediction of the flow stress in the post-necking ranges.

Figure 9 presents a comparison between the predicted loading forces from different hardening laws and the experimental data. The results indicate that all three models accurately predict the loading forces during the initial stages of deformation, up to the maximum force. However, beyond this point, the Swift model tends to overestimate the experimental data, while the Voce model underestimates it. In contrast, the LSV model shows an excellent prediction of the experimental results across wide deformation ranges. As seen in Equation (8), parameter α is used to adjust model’s prediction for the flow stress according to the forecasts of the Swift and Voce models, especially in the post-necking ranges. A larger α yields a higher prediction for the loading force after the maximum. Therefore, the value of this parameter can be determined by applying a searching algorithm, like the bisection method [41]. This study adopted this algorithm to identify parameter α of the LSV model.

The good agreement between the numerical prediction and the experimental data demonstrates the robustness and accuracy of the developed material model. Consequently, the calibrated LSV hardening law will be utilized in the subsequent section to predict the FLD of the tested material.

### 3.2. Theoretical Method for Forming Limit Diagram Prediction

#### 3.2.1. MMFC2 Method

The MMFC has been developed by Hora and co-authors [20] based on the observations of strain path changes occurring after the formation of the diffuse neck. The criterion for necking formation is mathematically expressed as follows:

(9) $$d\sigma_{1}=\frac{\partial \sigma_{1}}{\partial \varepsilon_{1}}d\varepsilon_{1}+\frac{\partial \sigma_{1}}{\partial \beta }\frac{\partial \beta }{\partial \varepsilon_{1}}d\varepsilon_{1}\geq \sigma_{1}d\varepsilon_{1}$$

where β is the strain increment ratio that governs the strain path evolution. Within the homogeneous deformation stages, i.e., $d\sigma_{1}>\sigma_{1}d\varepsilon_{1}$, the strain path is unchanged. Hence, $\delta \beta /\delta \varepsilon_{1}$ vanishes, and the MMFC model secures the Swift’s instability. Beyond diffuse necking, the strain path changes gradually toward the plane–strain deformation mode to achieve new stabilized states [20]. Therefore, the strain increment ratio is updated by the following equation:

(10) $$\Delta \beta =\frac{\sigma_{1}-\frac{\partial \sigma_{1}}{\partial \varepsilon_{1}}}{\frac{\partial \sigma_{1}}{\partial \beta }}\Delta \varepsilon_{1}$$

Then, the establishment of strain localization is achieved when the strain ratio approaches zero, i.e., β→0.

Pham et al. [25] recently highlighted the rapid β evolution predicted by the original MMFC model. Examined on a DP780 steel sheet, the theoretical estimation under the uniaxial tension is significantly faster than that derived from a corresponding FE analysis of the uniaxial tensile test. To address this discrepancy, a scaling factor, ξ, was introduced to decelerate the strain evolution predicted by the theoretical model. This modification is expressed mathematically as follows:

(11) $$\Delta \beta =\xi \frac{\sigma_{1}-\frac{\partial \sigma }{\partial \varepsilon_{1}}}{\frac{\partial \sigma_{1}}{\partial \beta }}\Delta \varepsilon_{1}$$

Their study recommended ξ=0.5 applying for several sheet metals to mimic the strain path changes observed during FE simulations. This study simply adopts the recommendation for the tested tubular material without any change.

**Figure 10:**
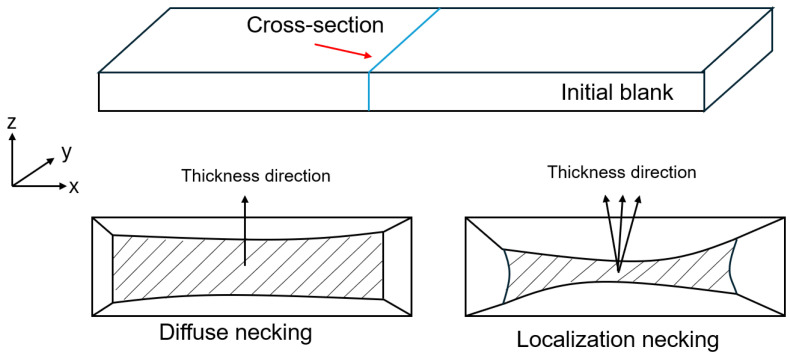
Concept of the out-of-plane deformation under necking condition in the MMMFC2 method [25].

Furthermore, a new condition for the initiation of strain localization was introduced by identifying out-of-plane deformation. Figure 10 illustrates the assumption of a change in the thickness direction at the onset of localized necking. This led to the development of an enhanced version of the MMFC, referred to as MMFC2. Owing to its superiority, this study examined the MMFC2 model in predicting the theoretical FLD of the tested C1100 pure copper tubes.

#### 3.2.2. MK Method

**Figure 11:**
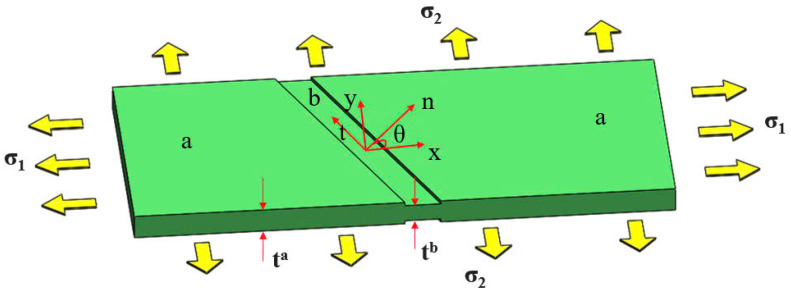
Illustration of the geometrical imperfection in the MK method.

The Marciniak–Kuzynski method [13,14] is a widely used method for predicting theoretically the FLD of sheet metals. This method was developed based on the observation that the inhomogeneity of the blank is unavoidable causing material imperfection. The imperfection is a consequence of different factors, for example, the surface roughness, and uniform distribution of chemical components over the blank. The MK method assumed a geometrical imperfection leveraging the material imperfection of the tested material, as shown in Figure 11. The material coordinates of the "safe" region (region a) differ from those of the grooved region (region b). Hence, the imperfection is initiated by a ratio between the thickness of region b, denoted by $t^{b}$, and the thickness of region a, denoted by $t^{a}$. The assumption states:

(12) $$f_{0}=\frac{t^{b}}{t^{a}}$$

Force equilibrium conditions on the transition between region a and region b yield:

(13a) $$\sigma_{nn}^{a}t^{a}=\sigma_{nn}^{b}t^{b}$$

(13b) $$\sigma_{nt}^{a}t^{a}=\sigma_{nt}^{b}t^{b}$$

In addition, the deform compatibility between these two regions requires the following equation:

(14) $$d\varepsilon_{tt}^{a}=d\varepsilon_{tt}^{b}$$

Given an increment of equivalent plastic strain, solving the system of Equations (12)–(14) provides the current state of deformation at the material point. During the deformation, the orientation of the groove is updated accordingly as follows:

(15) $$tan\left(\theta +\Delta \theta \right)=tan\left(\theta \right)\frac{1+d\varepsilon_{x}^{a}}{1+d\varepsilon_{y}^{a}}$$

Once the ratio between major strain increments of the two regions exceeds a critical value—for example, $\frac{\Delta \varepsilon_{1}^{b}}{\Delta \varepsilon_{1}^{a}}\geq 10$—the forming limit of the current loading condition is established. Furthermore, assigning different values of the initial groove’s angle associates different levels of the forming limit of the examined loading condition. Connecting the lowest level of the forming limits of all considering loading conditions constructs the predicted FLC. A MATLAB code is developed for this purpose.

## 4. Results and Discussion

This section presents and analyzes the theoretical FLDs predicted using the calibrated LSV hardening model with the von Mises yield function, employing both the MK and MMFC2 methods.

### 4.1. Comparison of FLD Predictions

**Figure 12:**
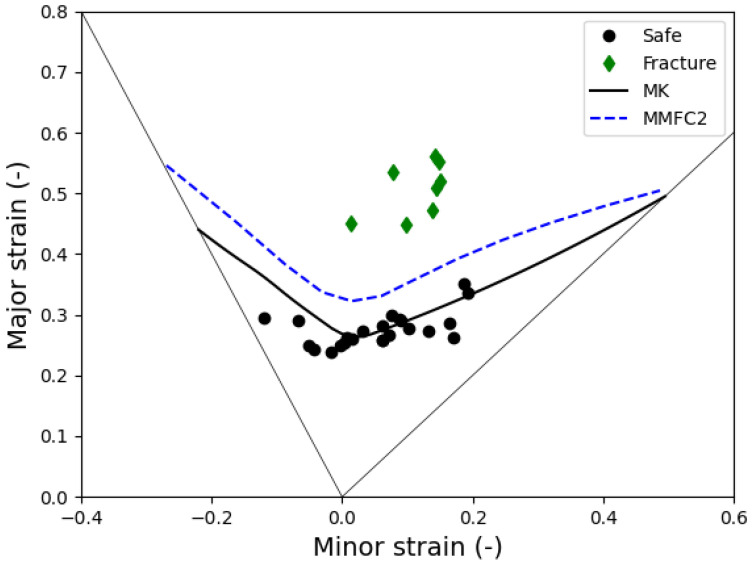
Comparison between the theoretically predicted forming limit diagram of C1100 copper and the experimentally measured data.

Figure 12 displays the predicted FLDs based on two methods, alongside experimental data measured in both the safe and fracture regions for comparison. As shown in the figure, the MK model predicts a more conservative FLD compared to the MMFC2 model. This finding contrasts with previous results reported for a CR4 steel sheet [25], where the predicted FLDs from both models showed similar levels of forming limits at the plane–strain tension, denoted as $FLC_{0}$. The observed difference emphasizes the importance of selecting an appropriate theoretical model for FLD prediction.

The experimental safe region data span from uniaxial to equi-biaxial tension, while fracture region data cluster between plane–strain and equi-biaxial tension. The MMFC2 model demonstrated superior accuracy in delineating safe and unsafe regions, especially under stretching conditions observed in tube hydroforming (THF). The MK model, while more conservative, provided reliable predictions in biaxial tension regions, validating its effectiveness when properly calibrated. This comparative analysis emphasizes the importance of incorporating strain path effects, as demonstrated by the MMFC2 model, for more accurate FLD predictions. Additionally, it highlights the adaptability of the MK model, which can be adjusted via imperfection parameters to refine predictions. Together, these insights contribute to advancing FLD prediction methodologies, providing valuable guidance for optimizing metal-forming processes involving C1100 copper tubes The integration of theoretical modeling with experimental validation advances FLD prediction methodologies for C1100 copper tubes. It also highlights the critical role of strain path considerations, particularly in THF processes. Future research should explore the impact of complex, non-linear strain paths on MMFC2 predictions to enhance its applicability in industrial forming processes.

Pham et al. [24] presented a rough estimation of $FLC_{0}$ based on the MMFC method, as follows:

(16a) $$k^{PS}=\frac{H^{\prime }\left({\bar{\varepsilon }}^{*}\right)}{H({\bar{\varepsilon }}^{*})}$$

(16b) $$FLC_{0}=\varepsilon_{1}^{*}=k^{PS}{\bar{\varepsilon }}^{*}$$

where $k^{PS}=1/\bar{\sigma }\left(1,\alpha \right)$ is the auxiliary function corresponding to the plane–strain stress state. Given the von Mises yield function, $k^{PS}=0.866$, for the plane–strain forming mode, in this equation, ${\bar{\varepsilon }}^{*}$ denotes the equivalent plastic strain of the material point at the instance of strain localization and $\varepsilon_{1}^{*}$ denotes the corresponding major strain. In an earlier study, Soare [42] proposed the conditions for determining the $FLC_{0}$ theoretically by solving the equations:

(17a) $$k^{PS}=\frac{H^{\prime }\left({\bar{\varepsilon }}^{b*}\right)}{H({\bar{\varepsilon }}^{b*})}$$

(17b) $$f_{0}=\frac{H({\bar{\varepsilon }}^{a*})}{H({\bar{\varepsilon }}^{b*})}\exp\left(\varepsilon_{1}^{b*}-\varepsilon_{1}^{a*}\right)<1$$

where superscripts ${\left(.\right)}^{a/b}$ denote the examined quantity of the region *a* and *b*, respectively. It can be observed that the solution of Equation (16) represents the upper limit of the solution for Equation (17), which is achieved when $f_{0}=1$ and ${\bar{\varepsilon }}^{a*}={\bar{\varepsilon }}^{b*}$. As a result, the MMFC2 method typically provides a higher prediction of the FLD compared to the MK method. However, the MK method offers greater flexibility in adjusting the level of the predicted FLD by modifying the value of the imperfection.

The comparative analysis of the MMFC2 and MK models reveals distinct predictive behaviors in forming limit diagram (FLD) estimation for C1100 copper tubes. The MMFC2 model generally predicts higher FLDs due to its advanced consideration of strain path evolution and out-of-plane deformation effects, making it particularly effective in capturing strain localization. In contrast, the MK model provides more conservative FLD predictions but offers greater adaptability through the adjustment of imperfection parameters, allowing for tailored applications in metal forming analysis. This study underscores the fundamental mathematical differences between the two models, highlighting MMFC2’s superiority in strain localization prediction while recognizing the MK model’s flexibility in accommodating material imperfections. Collectively, these models provide a robust framework for advancing theoretical FLD modeling in metal forming processes.

### 4.2. Application in Finite Element Analysis

**Figure 13:**
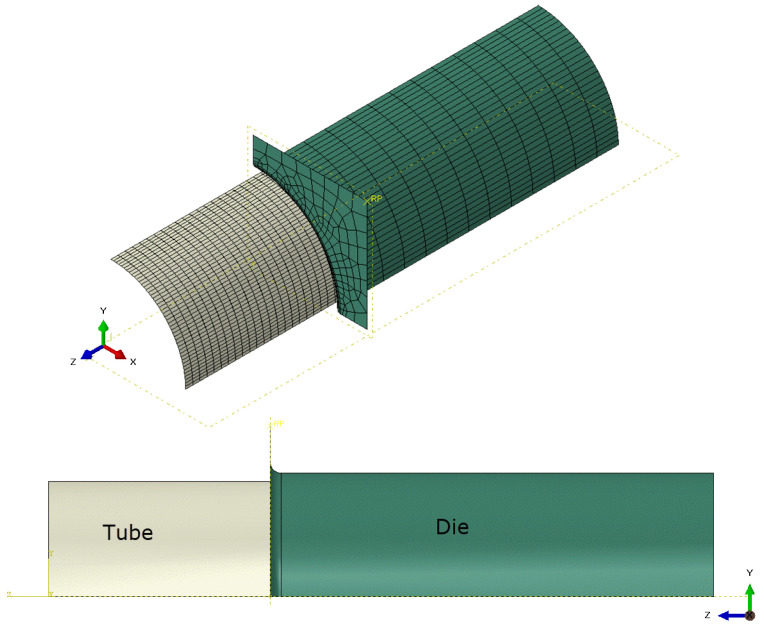
FE model for the tube expansion test.

To examine the application of the theoretical FLDs, an FE model is developed in Abaqus/Explicit software to simulate the tube expansion test. Due to the symmetry, only a quarter of the tube is modeled, as shown in Figure 13. The die is modeled by rigid body elements R3D4, while the tube is modeled by shell elements S4R. The mesh size in the longitudinal direction of the tube is 1 mm, while 45 elements were used to model the cross-section.

Similar to the experimental process, a ramp pressure of 18 MPa was applied on the inner surface of the tube. In addition, a displacement of 5 mm is assigned to the free edge of the tube. The calibrated LSV hardening law is employed together with the von Mises yield function to describe the plastic deformation of the tube. In addition, the predicted FLDs based on the MK and MMFC2 methods are applied to predict the failure of the tube using the ductile damage initiation option in the Abaqus/Explicit software. Damage evolution is controlled by a displacement of 0.01, based on the author’s experiences.

**Figure 14:**
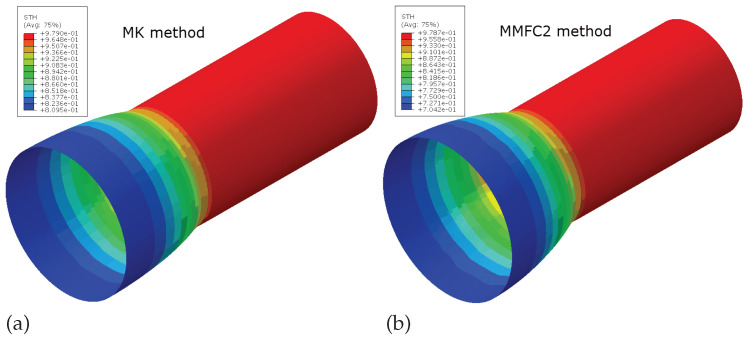
Prediction of the deformed tube before fracture: (**a**) MK method; (**b**) MMFC2 method.

Figure 14 displays the FE predictions of the thickness distribution along the deformed tube just prior to fracture. The MK model predicts failure earlier than the MMFC2 method. Consequently, the thickness and diameter of the deformed tube can be directly derived from these simulation results. Table 2 compares the predicted thickness and diameter, based on the two theoretical FLDs, with measurements from the safe region. This comparison reveals that the MK model anticipates failure sooner than observed experimentally, as reflected in the tube’s thickness and diameter. In contrast, the MMFC2 method’s FLD notably delays the predicted failure, as shown in Table 2. The actual measured thickness and diameter fall between the values predicted by the MK and MMFC2 models, suggesting that the true FLC of the tested material lies somewhere between these two theoretical curves. This encourages the use of the MMFC2 method for predicting FLDs of tubular materials, highlighting its practical potential.

## 5. Conclusions

This study compares the applications of the MMFC2 and MK methods in predicting the FLD of the C1100 pure-copper tube. Tube expansion tests were conducted to validate the accuracy of their theoretical predictions. The main findings after this work are as follows:

- Both the Swift and Voce hardening laws effectively capture the hardening behavior of the tested material observed during the uniaxial tensile test. However, both models provide unsatisfactory predictions for the hardening behavior in the post-necking ranges. Fortunately, the LSV model offers an excellent description of the hardening behavior of the C1100 copper tube.
- The calibrated LSV hardening law is used in the MMFC2 and MK models, coupled with the von Mises yield function, to predict the FLD of the tested material. The prediction made by the MMFC2 model is significantly higher than that of the MK model.
- A comparison between experimental and predicted FLDs demonstrates the practical value of the theoretical predictions. The predicted FLD proves to be a useful tool for evaluating failure due to necking that occurs during the hydroforming of the C1100 pure copper tube.
- The predicted FLDs are employed in the FEA of the tube expansion test. It is found that the MK model provides a conservative prediction of the failure of the tube. The prediction of the MMFC2 model is more relevant to the experimental measurements.

This research advances FLD prediction methodologies by integrating advanced material modeling with experimental validation. The findings underscore the significance of strain path effects in forming processes and demonstrate the superiority of the MMFC2 model for predicting necking behavior in tubular materials; while the current FLD predictions assume linear strain paths, future studies should address the influence of complex strain paths encountered in industrial tube THF processes. Further refinements of the MMFC2 model, including multi-path strain analysis, will enhance its applicability in real-world manufacturing scenarios.

## Figures and Tables

**Table 1:** Identified parameters of three hardening laws of the tested material.

Hardening Law	Swift			Voce			LSV
Parameter	*C* (MPa)	$\varepsilon_{0}$	*n*	*A* (MPa)	*B* (MPa)	*k*	α
Value	647.42	0.00236	0.472	393.52	333.52	6.73	0.45

**Table 2:** Comparison between the measurements and simulated geometries of the deformed tube in the safe region.

	Experiment				MK	MMFC2
	# 1	# 2	# 3	Average		
Thickness (mm)	0.75	0.78	0.76	0.763	0.809	0.704
Diameter (mm)	29.1	28.3	28.8	28.73	27.08	30.05

## Data Availability

The original contributions presented in this study are included in the article. Further inquiries can be directed to the corresponding authors.
